# Any resurgence of leprosy cases in the Togo’s post-elimination period? Trend analysis of reported leprosy cases from 2010 to 2022

**DOI:** 10.1186/s12879-024-09492-w

**Published:** 2024-06-16

**Authors:** Akila Wimima Bakoubayi, Falapalaki Haliba, Wendpouiré Ida C. Zida-Compaore, P’tanam P’kontème Bando, Yao Rodion Konu, Latame komla Adoli, Kodjo Akpadja, Kamevor Alaglo, Maweke Tchalim, P’niwè Patchali, Yaovi Djakpa, Komi Amekuse, Piham Gnossike, Denis A. Yawovi Gadah, Didier Koumavi Ekouevi

**Affiliations:** 1 German Leprosy and Tuberculosis Association, Lome, BP 2271 Togo; 2 Department of Public Health, Faculty of Health Sciences, University of Lome, Lomé, Togo; 3 African Center for Research in Epidemiology and Public Health, Lome, Togo; 4 National Tuberculosis Control Program, Lome, Togo; 5 National Program for Neglected Tropical Diseases, Lome, Togo

**Keywords:** Epidemiology, Leprosy, Spatiotemporal, Togo

## Abstract

**Background:**

Leprosy, or Hansen’s disease, is a chronic infectious disease caused by *Mycobacterium leprae*. Togo achieved the target of eliminating leprosy as a public health problem in 2000 (less than 1 case/10 000 population). However, new cases of leprosy are still being reported. The aim of this study was to describe and map trends of leprosy cases notified in Togo from 2010 to 2022.

**Methods:**

This was a descriptive cross-sectional study covering a thirteen-year period from January 1, 2010, to December 31, 2022. The data of the study were leprosy surveillance system’s data collected monthly between 2010 and 2022. The estimated number of leprosy cases and the incidence rate of leprosy cases were reported for the whole population by region, by district, by calendar year (2010–2022) and by target sub-population (children under 15, women and people with disabilities). Observed case incidence rates were mapped by health district and by year.

**Results:**

From January 1, 2010, to December 31, 2022, 1031 new cases of leprosy were diagnosed in Togo. The median age of subjects was 46 years (interquartile range: 33–60), with extremes from 4 to 96 years. Half the subjects were women (50.7%). Variations in the leprosy incidence rate by year show an increase between 2010 and 2022, from 0.7 cases /100,000 population to 1.1 /100,000 population respectively. From 2010 to 2022, the proportion of cases in children remained low, between 0 and 9%. The proportion of women fluctuated between 39.7% and 67.2% between 2010 and 2017, then stabilized at an average of 50% between 2018 and 2022. The proportion of multi-bacillary leprosy cases increased quasi-linearly between 2010 and 2022, from 70 to 96.6%. Mapping of leprosy cases showed that leprosy was notified in all Togo health districts during the study period, apart from the Lacs district, which reported no leprosy cases.

**Conclusion:**

Togo has achieved the elimination of leprosy as a public health problem. However, the increase in the number of new leprosy cases and the proportion of leprosy cases in children indicate that transmission of the disease is continuing and that supplementary measures are needed.

## Background

Leprosy, also known as Hansen’s disease, is a chronic infectious disease caused by *Mycobacterium leprae*, mainly affecting the skin and peripheral nerves [1]. The exact mode of transmission of leprosy is not yet known. Transmission is thought to occur via nasal droplets or prolonged skin contact with an untreated patient [2, 3]. Untreated or late-diagnosed patients develop irreversible disabilities and disfiguring complications [3]. These physical complications of leprosy are responsible for stigmatization and social exclusion [4].

In 1991, the World Health Assembly adopted a resolution to “eliminate” leprosy as a public health problem by the year 2000 [5]. Elimination, defined as an incidence rate of less than 1 case per 10 000 population/year, was achieved worldwide by the year 2000 in 107 countries (including Togo) out of 122 considered endemic in 1985 [6]. However, new cases of leprosy continue to be detected in all World Health Organization (WHO) regions [7]. In 2022, 174,087 new cases were reported worldwide, representing a detection rate of 21.8 cases per million inhabitants, an increase of 23.8% on 2021 (140,594 new cases). Most new cases were in the Southeast Asian region (66.5%), followed by the African region (15.1%). New cases among children indicate recent transmission [8]. In 2022, 10,302 new paediatric cases (5.9% of the total number of new cases) were reported worldwide, representing a rate of 5.1 cases per million children. The case detection rate of cases among children has risen by 14.6% compared to 2021 [7]. This means that leprosy persists, and its transmission continues in these different regions despite the availability of effective, free antibacterial treatment.

In Togo, the threshold for the elimination of leprosy as a public health problem (less than one case per 10,000 inhabitants/year) was reached in 2000 [6]. However, new cases of leprosy are still being reported. Togo, like other countries, has signed up to the WHO 2030 roadmap, which aims to eliminate leprosy (defined as interruption of transmission) by 2030 [9, 10]. To achieve this, it is important to review the evolution of leprosy over time and space, to know the current situation and to define or readjust control strategies to achieve the elimination (interruption of transmission) of leprosy. The present study aims to describe and map trends of leprosy cases notified in Togo from 2010 to 2022.

## Methods

### Study setting

Togo is a West African country with a total surface area of 56,785 km^2^ and a population of 8.09 million in 2022 [11].

**Fig. 1:**
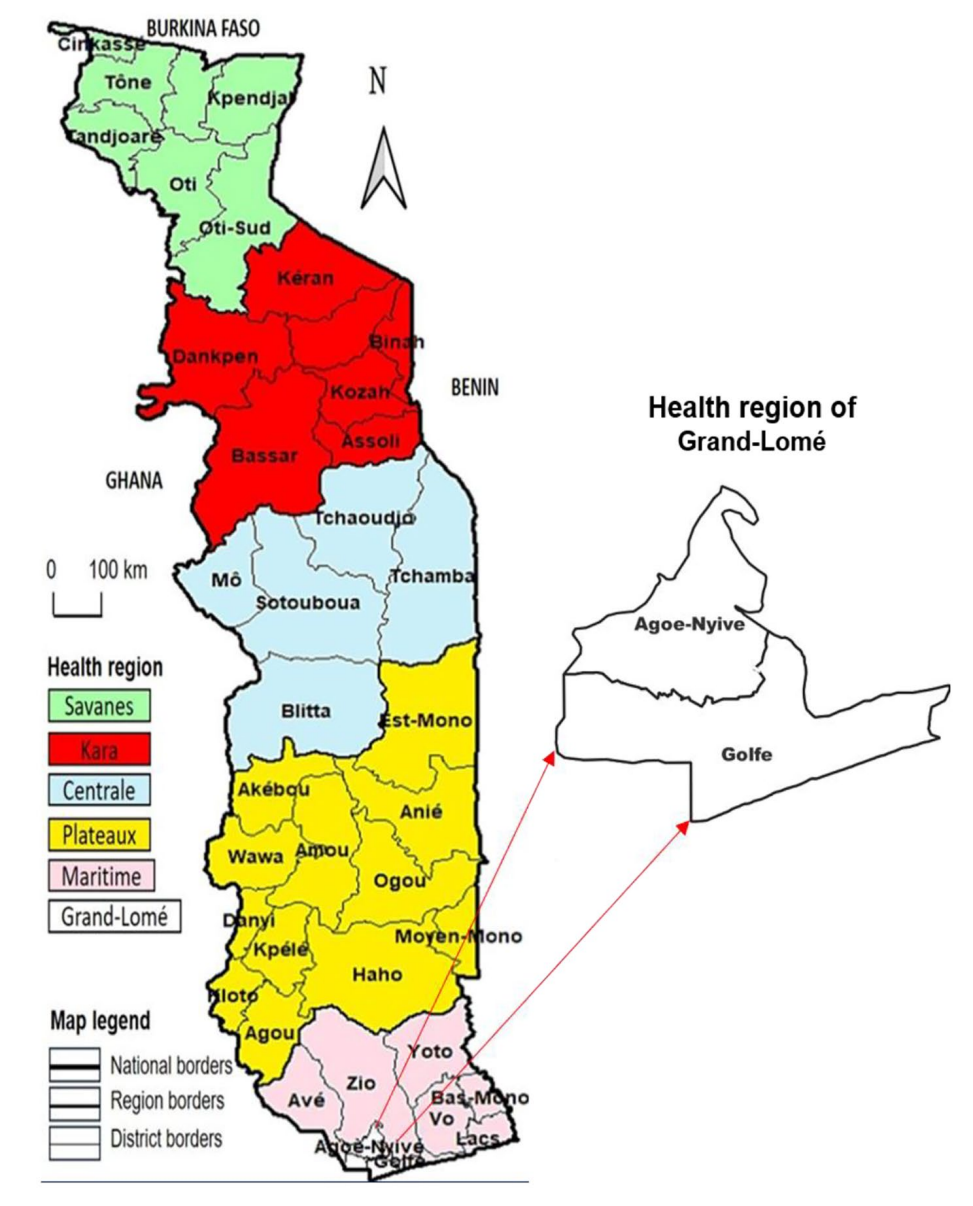
Maps of Togo’s health regions and districts

The Togolese healthcare system has a three-tier pyramid structure at three level: the central level, comprising the National Program for Neglected Tropical Diseases; the regional level, comprising the health regions; and the peripheral level, comprising the health districts and peripheral care units. Togo has six health regions (from north to south: Savana, Kara, Centrale, Plateaux, Maritime and Lomé-commune) and 39 health districts. An overview of Togo’s health regions and districts is provided in Fig. 1.

### Type of study

It was a descriptive cross-sectional study covering a thirteen-year period from January 1, 2010, to December 31, 2022.

### Data sources and data collection

The data analysed in this study were routine leprosy surveillance data collected monthly between 2010 and 2022 by the National Program for Neglected Tropical Diseases. Throughout this period, and in accordance with the guideline of the national neglected tropical diseases program, all clinically validated leprosy cases under treatment are systematically notified to the focal points of the 39 districts, which record these data in registers. The district focal points transmit these data to the regional level. These data are then compiled, checked, and validated at regional level before being transmitted to central level. National data validation meetings are held once a year, attended by all regional and district focal points for neglected tropical diseases, technical teams from the National Program for Neglected Tropical Diseases and non-governmental organizations involved in the fight against neglected tropical diseases. For the purposes of this study, data available at central level from 2010 to 2022 were extracted.

### Variables included

Variables collected for the study included demographic variables (age and sex of patients, regions and districts of patient residence) and clinical data: clinical forms of leprosy (pauci bacillary and multi bacillary), degree of disability of patients at the time of diagnosis according to WHO classification (0, 1, 2) of all new cases registered during the same period.

### Operational definitions

WHO classifies leprosy patients’ disabilities at the time of diagnosis into three degrees [12].

- Disability grade 0: No disability caused by leprosy in the eyes, hands, and feet.
- Disability grade 1: Eye problem caused by leprosy, but vision is not severely affected (equal to 6/60 or better; fingers can be counted from six meters); loss of sensitivity of hands or feet.
- Disability grade 2: Eyes: lagophthalmos and/or ectropion; trichiasis; visual impairment (fingers not counted at 6 m). Hands and feet: with visible lesions, i.e. clawed hands, drooping feet, relapses, etc. clawed hands, drooping feet, reabsorption of fingers.

Two clinical forms of leprosy are distinguished:

- Pauci-bacillary: Five or fewer lesions with no bacteria detected in the skin smear (sample taken from the area).
- Multi-bacillary: More than five lesions or detection of bacteria in the skin smear.

### Data processing and analysis

Numbers and incidence rates of leprosy cases were reported for the whole population by region, by district, by calendar year (2010–2022) and by target sub-population (children under 15, women and people with disabilities). Case incidence rates per 100,000 population were estimated as the ratio of the number of cases to the number of inhabitants. Observed case incidence rates were mapped by health district and year. Demographic data were provided by the Togo health statistics directory under the Ministry of Health.

### Ethical and regulatory aspects

This study was approved by the Bioethics Committee of the Togo’s Ministry of Health (No 025/2022/CBRS). Access to the database was authorized by the Ministry of Health through the National Program for Neglected Tropical Diseases. All data were anonymized, and confidentiality was strictly respected in data processing and analysis.

## Results

### Sociodemographic and clinical characteristics of leprosy patients

From January 1, 2010, to December 31, 2022, 1031 new cases of leprosy were diagnosed in Togo (Table 1). The median age of patients was 46 years (inter-quartile range: 33–60), with extremes from 4 to 96 years. Half of the patients were women (50.7%). Cases were diagnosed in all health regions of Togo (Plateaux 12.5% and Kara 12.0%). The majority (86.2%) of cases had multi-bacillary leprosy. At the time of diagnosis, 29.9% of cases had a grade 2 of disability.

**Table 1:** Sociodemographic and clinical characteristics of new leprosy cases in Togo from 2010–2022 (*N* = 1031)

	*n*	Proportion (%)
**Age (years)**		
Median [IQR]	46 [33–60]	
Mean (SD)	46 (17)	
Minimum-Maximum	4–96	
**Age groups (years)**		
< 15	38	3.7
≥ 15	993	96.3
**Gender**		
Female	523	50.7
Male	508	49.3
**Health region**		
Grand-Lome	70	6.9
Maritime	159	15.4
Plateaux	232	22.5
Centrale	154	14.9
Kara	227	22.0
Savanes	189	18.3
**Type of leprosy**		
Multibacillary	889	86.2
Paucibacillary	142	13.8
**Degree of disability**		
0	571	55.4
1	152	14.7
2	308	29.9

IQR = Interquartile Range; SD = Standard Deviation

### Trends in new leprosy cases

**Fig. 2:**
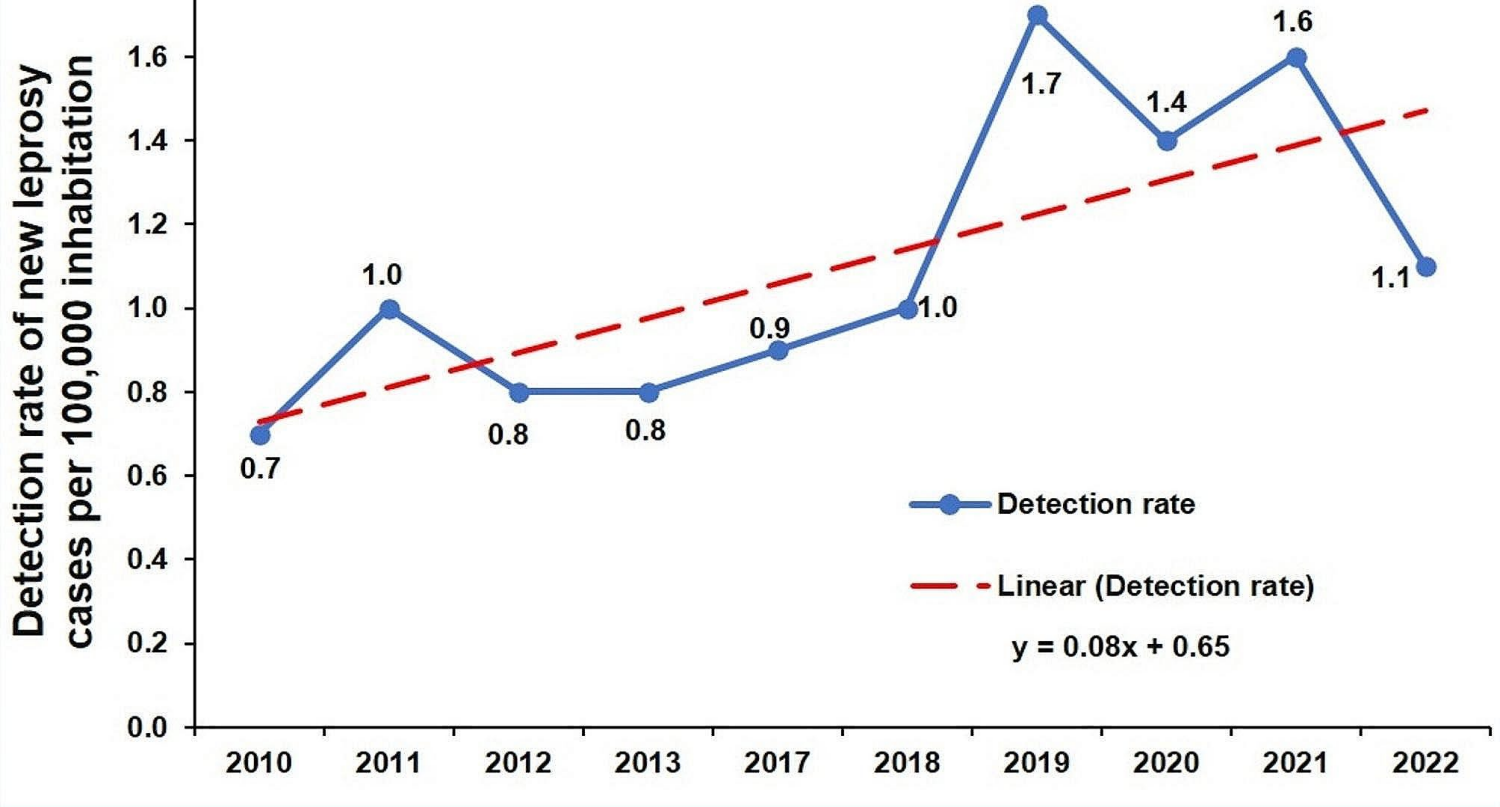
Evolution of the leprosy incidence rate in Togo, 2010–2022 (*N* = 1031)

Overall, since 2018, Togo has recorded at least 1 case per 100 000 population each year (Fig. 2). For the year 2022, Togo recorded 1.1 leprosy cases per 100,000 population at national level. Variations in the leprosy incidence rate by year show an increase between 2010 and 2022, from 0.7 cases /100,000 population to 1.1 /100,000 population respectively.

### Trends in new leprosy cases by health region

Over the study period, all health regions registered at least 1 case/100 000 inhabitants, apart from Greater Lomé, which recorded fewer than 1 case/100 000 inhabitant (0.3 cases per 100 000 inhabitants). The Central region and the Kara region registered at least 1 case/100 000 inhabitants each year (Table 2). Since 2018, the Maritime and Savana regions have registered more than 1 case of leprosy per 100,000 inhabitants each year. As for the Plateaux region, from 2010 to 2017 it recorded at least 1 case of leprosy each year and since 2018 less than 1 case each year.

**Table 2:** Cases of leprosy in Togo, 2010–2022: Number and rate per 100 000 inhabitants for each health region (*N* = 1031)

Heath regions		2010	2011	2012	2013	2014	2015	2016	2017	2018	2019	2020	2021	2022	Annual trends		
															Number and average rate	IC 95%	P-value
**Grand Lome**	Number	0	0	0	0	8	8	9	7	5	10	12	6	5	**70**		
	Rate	0.0	0.0	0.0	0.0	0.4	0.4	0.5	0.4	0.3	0.5	0.6	0.3	0.2	**0.3**	0.1–0.4	0.001
**Maritime**	Number	6	2	1	2	20	15	8	11	16	16	17	25	20	**159**		
	Rate	0.6	0.2	0.1	0.2	1.8	1.3	0.7	0.9	1.3	1.3	1.3	1.9	1.5	**1.0**	0.6–1.4	< 0.001
**Plateaux**	Number	16	29	26	28	21	28	22	14	9	10	11	13	5	**232**		
	Rate	1.3	2.3	2.0	2.1	1.5	2.0	1.5	1.0	0.6	0.7	0.7	0.8	0.3	**1.3**	0.9–1.7	< 0.001
**Centrale**	Number	7	12	9	9	12	10	6	9	12	26	17	14	11	**154**		
	Rate	1.2	1.9	1.4	1.4	1.8	1.5	0.9	1.3	1.7	3.5	2.2	1.8	1.4	**1.7**	1.3–2.1	< 0.001
**Kara**	Number	14	19	14	8	7	10	12	11	14	35	19	36	28	**227**		
	Rate	1.9	2.5	1.8	1.0	0.9	1.2	1.4	1.3	1.6	3.8	2.0	3.7	2.8	**2.0**	1.4–2.6	< 0.001
**Savana**	Number	0	0	1	4	13	12	7	16	19	29	34	36	18	**189**		
	Rate	0.0	0.0	0.1	0.4	1.4	1.2	0.7	1.6	1.8	2.7	3.1	3.2	1.6	**1.4**	0.7-2.0	0.001

IC = confidence interval

### Trends in new leprosy cases by target population (children under 15. Women, subjects with multi-bacillary leprosy and subjects with disability)

**Fig. 3:**
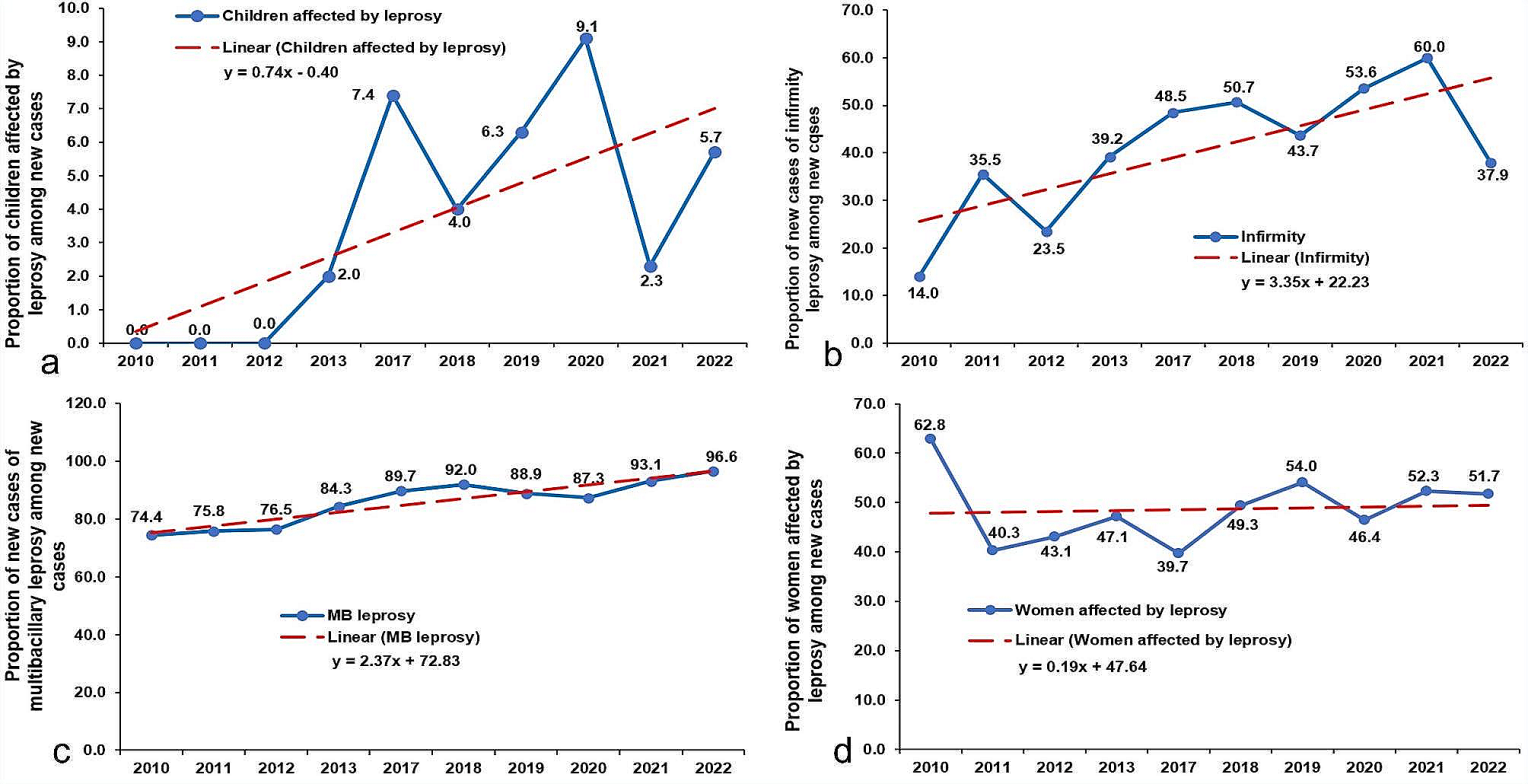
Trends in new leprosy cases by target population in Togo, 2010–2022: (**a**) Proportion of children affected by leprosy among new cases; (**b**) Proportion of persons with infirmity among new cases; (**c)** Proportion of multi-bacillary leprosy among new cases; (**d**) Proportion of women affected by leprosy among new cases. MB = multibacillary

The proportion of cases among children has generally remained low, between 0% and 9.1%. The proportion of leprosy cases among children by year shows an increase between 2010 and 2022, from 0 to 5.7%. However, this dynamic was not linear, with a sawtooth pattern. The proportion of females fluctuated between 39.7% and 67.2% between 2010 and 2017. It remained stable with an average of 50% between 2018 and 2022 (Fig. 3).

Among new leprosy cases detected, the proportion of multi-bacillary leprosy cases was relatively high throughout the period, with an almost linear increase between 2010 and 2022 from 70 to 96.6%.

The proportion of new cases with disability increased almost linearly from 14% in 2010 to 37.9% in 2020, with a peak of 60% in 2021.

### Mapping of observed leprosy cases by health district and year

The mapping of leprosy cases by district and year showed that, apart from the Lacs district, which recorded no leprosy cases over the entire study period, leprosy was diagnosed in all Togo’s health districts.

The number of health districts affected by leprosy rose from 10 in 2010 to 26 in 2022, with variations from one year to the next. More than 2 cases of leprosy per 100 000 population have been recorded in the districts of Kéran, Binah, Bassar and Avé each year over the last 4 years (2019–2022).

**Fig. 4:**
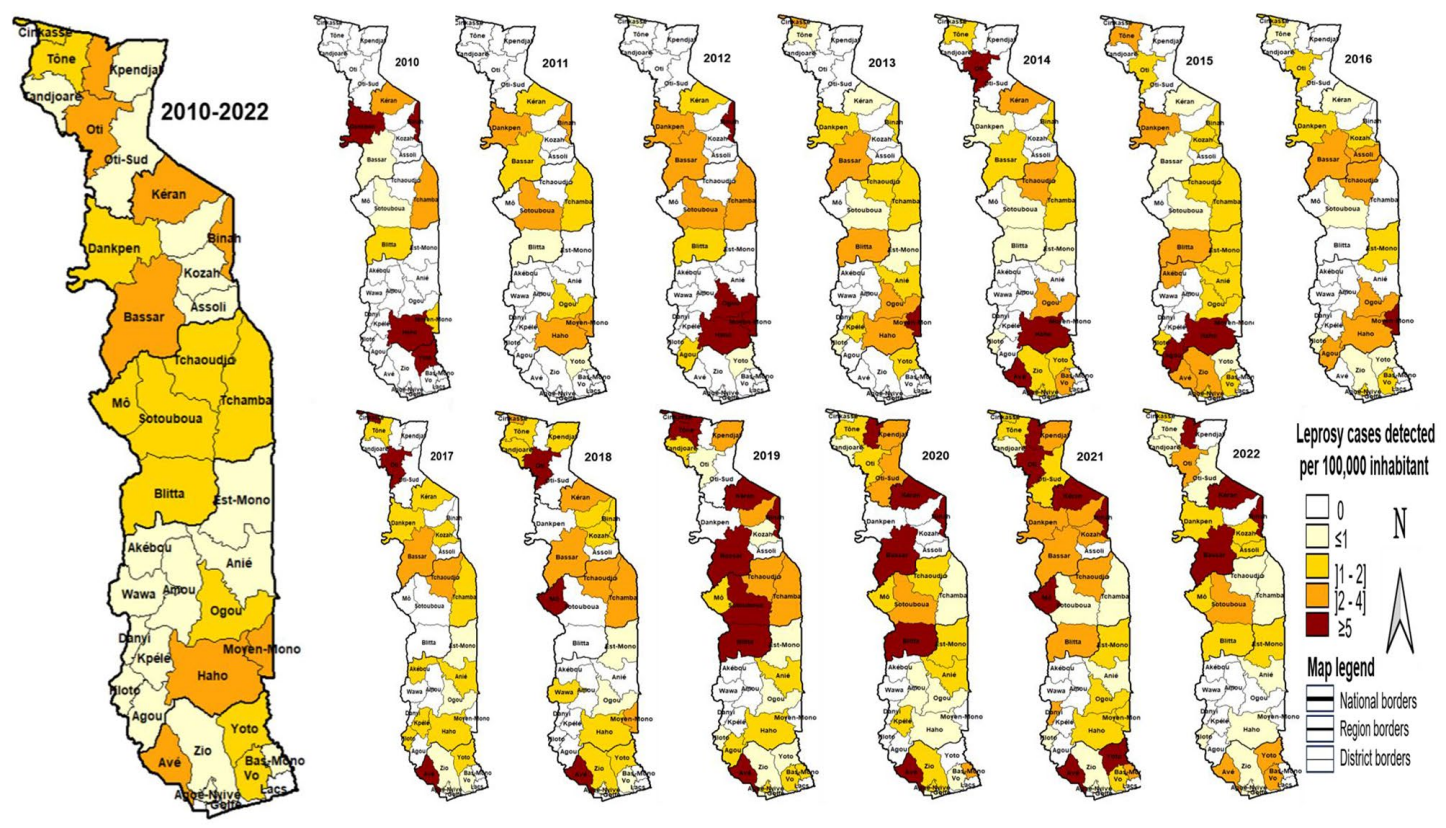
Map of new leprosy cases by health district in Togo, 2010–2022

Over the same period, the districts of Mô and Blitta recorded more than 1 case of leprosy per 100,000 population. The mapping of leprosy cases recorded during the study period by health district and year is presented in Fig. 4.

## Discussion

This study uses the completed database of the national program of neglected tropical diseases. It enabled us to provide a spatiotemporal description of leprosy cases registered in Togo between 2010 and 2022. The median age of patients was 46 years. Between 2010 and 2022, the incidence rate rose from 0.7 cases/100 000 population to 1.1/100 000 population. In 2022, Togo recorded 9.1% of leprosy cases in children under 15. The proportion of multi-bacillary leprosy cases increased almost linearly between 2010 and 2022, rising from 70 to 96.6%. The proportion of new cases with disability was 37.9% in 2022, with a peak of 60% in 2021. Mapping leprosy cases by district and year showed that, apart from the Lacs district, which registered no leprosy cases over the study period, leprosy was reported in all Togo’s health districts. The number of health districts affected by leprosy rose from 10 in 2010 to 26 in 2022, with variations from one year to the other.

The incidence rates observed confirm the elimination of leprosy as a public health problem in Togo. The elimination of leprosy, defined as an incidence rate of less than 1 case per 10,000 population /year, was achieved in Togo since 2000 [6]. However, new cases of leprosy continue to be detected. Variations in the national leprosy incidence rate by year show an increase between 2010 and 2022. This means that leprosy persists, and its transmission continues despite the progress made in the fight against the disease. The study we have conducted, based on data collected at district level, does not allow us to explain this increase. However, the increased competence of health personnel in leprosy diagnosis and the strengthening of awareness-raising and screening activities in recent years could explain the increase in new leprosy cases observed. The jagged reporting of leprosy cases may be explained by the discontinuity of funding because funding for leprosy control activities in Togo comes mainly from external sources.

In our study, both sexes were equally affected (50.7% in women and 49.7% in men). This result is contrary to what is frequently described in the literature. A study realized in South-East Ethiopia found a male predominance of 64.5% [13]. In Nigeria, a similar study found a male/female sex ratio of 2.1 [14]. This male predominance may be partly explained by the fact that men have easier access to health services in rural area in Togo [15].

The mean age of patients at diagnosis was 46 years. Kabo et al. in Chad and Gnimavo .al. in Benin reported a mean age of 38 years [16, 17]. Martins et al. in Brazil reported an average age of 47 [18]. Leprosy is therefore mainly reported in young people. Thus, leprosy in this segment of the population can have negative repercussions on the individual, his or her family and the country’s economy in the long term [18, 19]. This also implies transmission at younger ages, therefore probably transmission is still a problem.

The geographical distribution of cases highlights the elimination of leprosy as a public health problem at sub-national level. Cumulatively over the entire study period, all health regions recorded at least 1 case per 100 000 inhabitants, apart from Grand Lome (Togo’s capital), which recorded 0.3 cases per 100 000 inhabitants. This difference may be explained by limited access to drinking water and/or ineffective hygiene measures outside the capital, which are important determinants for the occurrence of leprosy [20]. These factors alone cannot explain this difference. Indeed, limited access to care outside the capital Lome and the way in which cases are detected may also explain this difference. It would therefore be important to initiate prospective studies to identify all the factors associated with the unequal distribution of leprosy in other health regions outside the capital Lome.

In the present study, we also noted that 96.3% of patients were aged 15 and over. Children (under the age of 15) accounted for only 3.7% of patients. These findings are like those of studies carried out by Ghunawat et al. in Dehli. Wangara et al. in Kenya and Gnimavo et al. in Benin, who found that children’s leprosy cases accounted for 7.1%, 7.5% and 10.1% of total recorded leprosy cases [17, 21, 22]. An increase in the proportion of cases in children under 15 years of age from 0 to 5.7% was observed in our study. As the proportion of children is considered an indicator of recent transmission of the disease, the increase observed in this study period is evidence that, until 2022, there was persistent transmission of the infection in the communities.

Our results show that multi-bacillary forms were the most frequent (86.3%). A high proportion of multi-bacillary leprosy cases has also been reported in other studies in Benin, Brazil, and China [17, 23, 24]. The high proportion of multi-bacillary forms increases the risk of contagiousness of the disease as well as the duration of treatment, and may lead to patients abandoning treatment, thereby contributing to the spread of the disease and the development of resistant forms [25]. Particular attention must be given to monitoring these indicators (loss of follow up, re-treatment, resistance cases) in Togo.

The proportion of new cases with disability was 37.9% in 2022. This proportion was 32% in Benin in 2018 and 19.1% in Chad in 2019 [16, 17]. This result indicates late diagnosis of leprosy and/or delayed management with multidrug therapy. High disability rates have a negative socio-economic impact on the communities [26]. In Togo, this delay in diagnosis is due to a lack of knowledge of the signs of the disease by the patients and health professionals, and to the stigma attached to leprosy patients. The commitment of countries to ensure early diagnosis, institute multidrug therapy and combat stigma has been suggested as a means of reducing the psychosocial and economic impact of leprosy [27]. Therefore, It is possible that the adoption of similar strategies could have an impact on reducing disability in highly endemic communities in Togo.

One of the limitations of this study is its retrospective nature, based on data collected at district level. An analysis of data quality shows missing data on management cases, follow-up cases, treatment failures, repeat treatments, resistance cases and side-effects management. These indicators provide information on the quality of screening, treatment, and follow-up cases. The availability of those information should enable us to carry out a holistic analysis of all the key indicators as defined by the WHO. The National Program for Neglected Tropical Diseases needs to reflect on how to improve the quality of data collection and use digital tools for data reporting. Another limit is the type of case detection which may partly explain the trends observed. In Togo, leprosy cases finding is passive in routine. However, as, and when the national program for neglected tropical diseases receives financial support from a partner, active integrated case finding activities are implemented over the funding period. Given the study period and the changes in the program’s staff, it was not possible for us to trace all the projects that have enabled active case-finding and their duration of execution since 2010, to establish a link with the trends observed.

## Conclusion

Togo has achieved the elimination of leprosy as a public health problem according to the WHO definition. However, the increase in the number of new leprosy cases, the predominance of multi-bacillary forms, the proportion of leprosy cases in children and the high number of grade 2 disabilities demonstrate that transmission of the disease is continuing. Additional measures such as strengthening the capacity of health personnel to diagnose leprosy, raising public awareness of the early signs of the disease and combating the stigmatization of leprosy patients are needed to achieve the WHO goal of eliminating (interrupting transmission of) leprosy by 2030.

## Data Availability

The datasets used and/or analysed in the current study are available from the corresponding author upon request with valid justification.

## Abbreviations

WHO: World Health Organization
IQR: Interquartile Range
SD: Standard Deviation
IC: Confidence Interval
MB: Multibacillary
